# Duodenum-Preserving Resection of the Head of the Pancreas: The Significance as a Diagnostic Therapy for the Lesion in the Pancreatic Head

**DOI:** 10.1155/1996/91608

**Published:** 1996

**Authors:** Y. Watanabe, M. Sato, T. Lee, Y. Abe, Y. Nakata, H. Kashu, S. Iseki, Y. Mizukami, S. Kimura

**Affiliations:** 1 Second Department of Surgery University of Ehime Shigenobu Ehime Shitsukawa 791-02 Japan; 2 Third Department of Internal Medicine Shigenobu Ehime Shitsukawa 791-02 Japan

## Abstract

A 75-year-old man who was diagnosed as having mucin-producing pancreatic cystic lesion ofthe main pancreatic duct by duodenoscopic examination was reported. Because of the low malignant potential of such lesions, duodenum-preserving resection of the head of the pancreas was performed, and the intra-operative histological examination showed no malig-nancy of the resected pancreatic head and no other surgical procedures, such as lymph-adenectomy nor pancreato-duodenectomy were necessary. The significance of this case report lies in that a less invasive operation should be selected at first to diagnose whether the lesion is malignant or not, and als0 that the selected operation itself must be sufficient to resect an adequate part of the pancreatic tissue involving the cystic lesion, ifnot malignant. Here, we report the process to select the procedure and the surgical technique.


*HPB Surgery*, 1996, Vol. 9, pp. 107–111
Reprints available directly from the publisher
Photocopying permitted by license only

© 1996 OPA (Overseas Publishers Association)
Amsterdam B.V. Published in The Netherlands
by Harwood Academic Publishers GmbH
Printed in Malaysia

CASE REPORT

### Duodenum-Preserving Resection of the Head of the Pancreas: The Significance as a Diagnostic Therapy for the Lesion in the Pancreatic Head

Y. WATANABE, M. SATO, T. LEE, Y. ABE, Y. NAKATA, H. KASHU, S. ISEKI, Y. MIZUKAMI* and S. KIMURA

Second Department of Surgery, University of Ehime, *Third Department of Internal Medicine, Shigenobu, Shitsukawa, Ehime 791-02, Japan

(*Received May 2, 1994*)

A 75-year-old man who was diagnosed as having mucin-producing pancreatic cystic lesion of the main pancreatic duct by duodenoscopic examination was reported. Because of the low malignant potential of such lesions, duodenum-preserving resection of the head of the pancreas was performed, and the intra-operative histological examination showed no malig-nancy of the resected pancreatic head and no other surgical procedures, such as lymph-adenectomy nor pancreato-duodenectomy were necessary. The significance of this case report lies in that a less invasive operation should be selected at first to diagnose whether the lesion is malignant or not, and also that the selected operation itself must be sufficient to resect an adequate part of the pancreatic tissue involving the cystic lesion, if not malignant. Here, we report the process to select the procedure and the surgical technique.

KEY WORDS: Mucin-producing pancreatic cystic lesion duodenum-preserving resection of the head of the pancreas.

### INTRODUCTION

For mucin-producing pancreatic cystic lesion there is in controversy as to the differential diagnosis between cystadenoma or cystadenocarcinoma and thus, the indication for operation and operative procedure are difficult to select. Once diagnosed as having a cystic lesion in the pancreas, operation should be selected, in most cases. But the operative procedure must be selected according to the degree of malignancy, the spread of the lesion and the patient's physical status. Operations such as conventional pancreatoduodenectomy (PD), pylorus-preserving pancreatoduodenectomy (PPPD)[1] and duodenum-preserving resection of the pancreas head (DPRPH)[2] are the options to be selected for a lesion in the head of the pancreas. However, the operative risk, morbidity and mortality decrease in the order as described above. So, the selected surgical procedure should be less invasive and safer, if the pancreatic lesion is not diagnosed as malignant, preoperatively.

Here, we report a case diagnosed as having a mucin-producing pancreatic cystic lesion, who was diagnosed and treated by DPRPH. This article deals with the surgical technique of the DPRPH and the process to select the procudure.

### CASE REPORT

A 75-year-old man initially presented with right hypochondric pain due to gall stones in August, 1993. He

*Address for correspondence:* Mr. Y. Watanabe, Second department of surgery, Ehime University School of Medicine, Onsen-gun, Shitsukawa, Shigenobu-cho 791-02, Japan.

was admitted to the hospital with a diagnosis of gall stones and an endoscopic retrograde cholangio-pancreatography (ERCP) was performed to rule out the presence of a common bile duct stone. ERCP on September 10, 1993 revealed a little stenotic main pancreatic duct and cyst formation in the main pancreatic duct about 15 mm in diameter with mucin formation in it (Fig. 1). Cytology of the mucin in the pancreatic duct, which was taken on October 26, 1993, showed only atypical columnar cells (Class II), however, biopsy from the pancreatic duct beside the cystic lesion showed papillary growth of the pancreatic ductal mucosa (Grade III). Computed tomography (CT) and angiography showed no abnormal findings, however, ultrasound (US) and endoscopic ultrasound (EUS) showed obscure cyst formation in the pancreatic head. Laboratory data, such as liver function test or pancreatic enzymes were in the normal range. Thus, the diagnosis was a mucin-producing cystic lesion in the pancreas head with a suspicion of malignancy. Surgical procedures, such as conventional PD, PPPD and DPRPH, were discussed and it was determined that DPRPH was to be performed for diagnostic assessment & therapy if the lesion was not malignant according to the intra-operative pathological examination. If necessary, for a malignant lesion, further PPPD or PD with lymphadenectomy was scheduled. All of these processes were discussed with the patient and his family and they agreed with this therapeutic program.

### OPERATIVE PROCEDURE

On November 24, 1993, the operation was performed. At laparotomy, no mass around the pancreatic head was palpable and there was no evidence of pancreatic cancer. Cholecystectomy with the resection of the

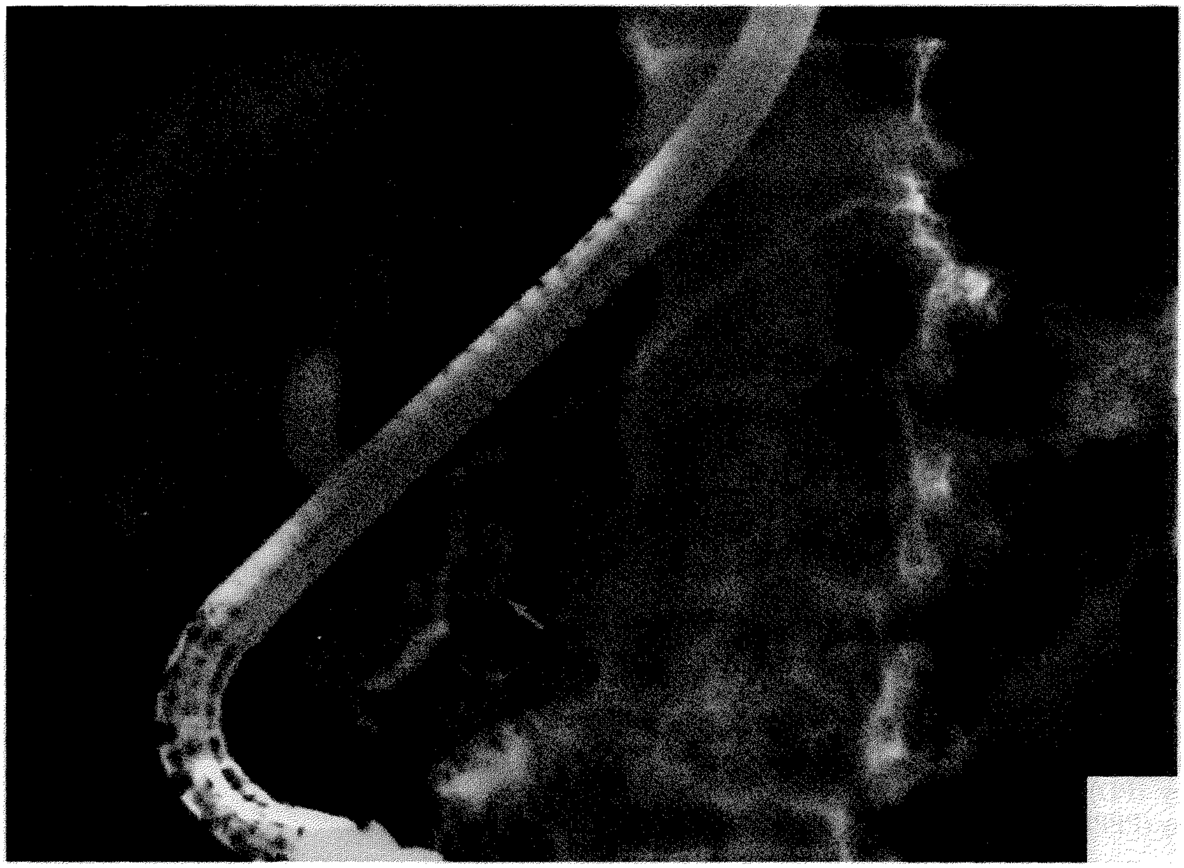

**Figure 1** ERCP demonstrating cyst formation (shown by arrow) in the main pancreatic duct about 15 mm in diameter with mucin formation in it.

common bile duct were performed (Fig. 2). The gastroduodenal artery, anterior inferior pancreatic arteries and the branches were cut just along medial wall of the duodenum. The main pancreatic (Wirsung) and accessory pancreatic duct were observed easily and doubly ligated and thus, resection of the pan-creatic head was completed (Fig. 3a). Intra-operative pathological examination showed no malignancy and thus, no other surgical procedures were performed. The stump of the pancreas was anastomosed with the jejunum and intestinal reconstruction was by Roux-en Y anastomosis (Fig. 3b).

Mobilization of the duodenum was not performed to avoid cutting the important blood supply to the duodenum, which seemed a very important blood supply after cutting the gastroduodenal artery and anterior inferior pancreatic arteries. However, after the resection of the pancreatic head, the duodenum (5 cm from the pyloric ring towards the third portion) appeared a little pale, indicating a reduced blood supply to the duodenum.

### POSTOPERATIVE COURSE

No complications nor complaints were seen, postoperatively. An H2-blocker was administered twice a day to prevent a peptic ulcer due to the decreased blood supply to the duodenum. On the 13th day after the operation, he started to eat, however, the H2-blocker was also taken twice a day. Most laboratory data were normal except a pancreatic exocrine function test estimated by the Pancreatic Function Diagnostant (PFD) test of 10.7% (normal range > 70%) just before discharge from the hospital. He could eat three meals a day, but did not suffer from fat diarrhea. Body weight loss in his hospital stay was 1.4 Kg.

He was discharged from the hospital on January 13, 1994. Three months later, no ulcers in the stomach nor duodenum were observed gastroduodenoscopically.

**Figure 2** Resected pancreas head with cyst formation (shown by arrow) and gall bladder containing two gall stones. See Color Plate II.

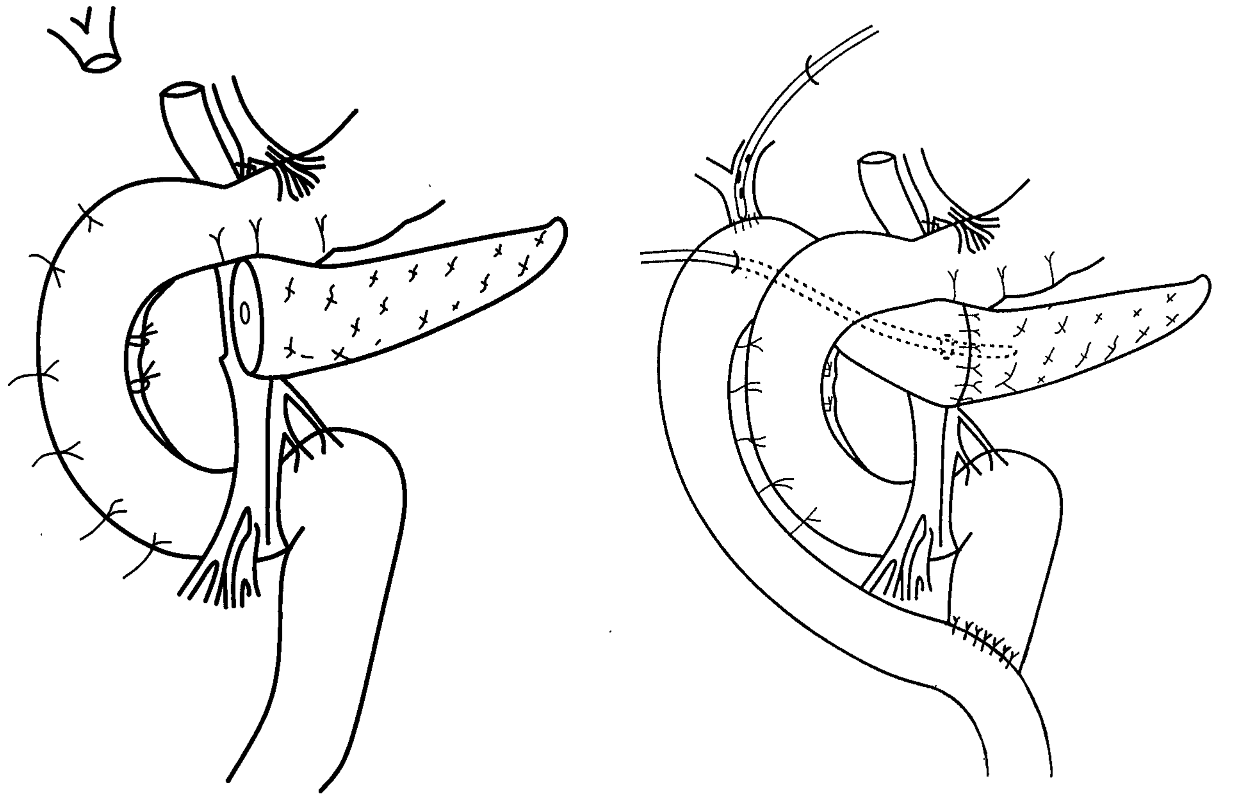

(a) (b)

**Figure 3** Figures demonstrating the operation method. a) Diagram of the resected area. Main and accessory pancreatic ducts are ligated along the duodenum. Mobilization of the duodenum was not performed to preserve the blood supply. Ligation of the gastroduodenal artery and anterior inferior pancreatic artery were performed, with intact blood supply from the posterior inferior pancratic artery. b) Reconstruction with pancreatojujunostomy, end-to-side choledochojejunostomy and end-to-side jejunojejunostomy. RTBD tube and pancreatic drainage tube were placed as shown in the diagram.

### DISCUSSION

Mucinous pancreatic cystic lesion is an uncommon disease of the pancreas. Recently, several cases have been reported and classification attempted. Suyama *et al.*[3], Yanagisawa *et al.*[4], and Itai and Ohhashi[5] suggested a new type of mucin-producing cystic tumor, characterized by localized cystic dilatation of the pancreatic duct. However, it is still obscure and difficult to distinguish the mucin-producing cystic tumor from the classic type of mucinous cytic tumor and mucinous duct ectasia[6]. Our case reported here can be classified as a typical mucin-producing cystic tumor, which was finally diagnosed histologically, from the resected pancreatic head, as an atypical hyperplasia.

A cystic lesion and its malignant counterpart (mucinous cystadenocarcinoma, for instance) of the pancreas are still not easy to distinguish from each other without a histological examination. In fact, the latent malignancy of this disease has been reported and the resection seems soundly based[7]. So, the strate-gy for this disease should be as follows: 1) resection of the lesion to diagnose whether the lesion is malignant or not, 2) if the lesion is not malignant, duodenum and the pancreatic tissue should be preserved as much as possible, 3) if a malignant lesion is found, further procedure, such as the conventional PD or PPPD should be selected. For the exclusion of malignancy and as a precaution against malignant change of the lesion, we recommend the DPRPH for resection of the pancreatic head involving the cystic lesion for intra-operative histological examination and then further appropriate procedures for malignancy.

DPRPH is an operation introduced by Beger[2] for pancreatitis. The advantages of this procedure are that the stomach and duodenum are preserved and the risks of the operation are lower than other procedures. They should be used for such cases reported in this article, that are difficult to diagnose as malignant or not before the operation. In conclusion, DPRPH can be safely performed and can be a reliable diagnostic therapy.

In this case, gastroduodenal artery and anterior inferior pancreatic artery were cut and posterior inferior pancreatic artery was preserved. The mobilization of the duodenum was not performed to avoid cut tiny the important blood supply to the duodenum, which seemed a very important after cutting the gastroduodenal artery and inferior pancreatic arteries. However, after the resection of the pancreatic head, the duodenum (5 cm from the pyloric ring towards the third portion) appeared a little pale, meaning a reduced blood supply to the duodenum. If the lesion is not malignant, pancreatic arterial arcades should be preserved, as described by Beger, however, the purpose of our surgical procedure was a total resection of the pancreatic head because of the probability of malignancy. The color of the duodenum was a little pale during the operation and 3 weeks after the operation, an ulcer was observed between the second and third parts of the duodenum by duodenoscopic examination. The ulcer was easily treated with on H2 blocker and ascertained to be cured 3 months after the operation.

Thus, this procedure should be the first option not only for the chronic pancreatitis, but also for the cystic lesion of the pancreatic head if the lesion is not malignant.

### REFERENCES

1. Traverso, L. W. and Longmire, W.P. (1978) Preservation of the pylorus in pancreaticoduodectomy. *Surg. Gynecol. Obstet.*, **146**, 959–962.
2. Beger, H. G., Krautzberger, W., Bittner, R., Buechler, M. and Limmer, J. (1984) Duodenum-preserving resection of the head of the pancreas in patients with severe chronic pancreatitis. *Surgery*, **97**, 467–473.
3. Suyama, M., Ariyama, J. and Shimaguchi, S. (1985) Clinicopathology of mucin-producing cancer of the pancreas. *Diagn. Imaging. Abdomen.*, **5**, 289–296.
4. Yanagisawa, A., Katoh, Y., Sugano, H., Takekoshi, T. and Takagi, K. (1984) Classification of the pancreatic cyst with atypical epithelium. *Biliary Tract Pancreas*, **5**, 1079–1085.
5. Itai, Y. and Ohhashi, K. (1986) Ductectatci mucinous cystadenoma and cystadenocarcinoma of the pancreas. *Radiology*, **161**, 697–700.
6. Bastid, C., Bernard, J. P., Sarles, H., Payan, M. J. and Shel, J. (1991) Mucinous ductal ectasia of the pancreas: A premalignant disease and a cause of obstructive pancreatitis. *Pancreas*, **6**, 15–22.
7. Tian, F., Myles, J. and Howard, J. M. (1992) Mucinous pancreatic ductal ectasia of latent malignancy: An emerging clinicopathologic entity. *Surgery*, **111**, 109–113.

